# Erlotinib ‘dosing-to-rash’: a phase II intrapatient dose escalation and pharmacologic study of erlotinib in previously treated advanced non-small cell lung cancer

**DOI:** 10.1038/bjc.2011.332

**Published:** 2011-08-30

**Authors:** A C Mita, K Papadopoulos, M J A de Jonge, G Schwartz, J Verweij, M M Mita, A Ricart, Q S-C Chu, A W Tolcher, L Wood, S McCarthy, M Hamilton, K Iwata, B Wacker, K Witt, E K Rowinsky

**Affiliations:** 1 Institute for Drug Development, Cancer Therapy and Research Center, University of Texas Health Science Center, 4th Floor, 7979 Wurzbach Road, Zeller Building, San Antonio, TX 78229, USA; 2 Erasmus University Medical Center, Postbus 2040, Rotterdam, 3000 CA, The Netherlands; 3 Brooke Army Medical Center, 3851 Roger Brooke Drive, Fort Sam Houston, TX 78234, USA; 4 OSI Pharmaceuticals, 2860 Wilderness Pl, Boulder, CO 80301, USA

**Keywords:** erlotinib, non-small cell lung cancer, EGFR, skin rash

## Abstract

**Background:**

To evaluate the anticancer activity of erlotinib in patients with previously treated, advanced non-small cell lung cancer (NSCLC) whose dose is increased to that associated with a maximal level of tolerable skin toxicity (i.e., target rash (TR)); to characterise the pharmacokinetics (PK) and pharmacodynamics (PD) of higher doses of erlotinib.

**Methods:**

Patients initially received erlotinib 150 mg per day. The dose was successively increased in each patient to that associated with a TR. Anticancer activity was evaluated. Plasma, skin, and hair were sampled for PK and PD studies.

**Results:**

Erlotinib dose escalation to 200–475 mg per day was feasible in 38 (90%) of 42 patients. Twenty-four (57%) patients developed a TR, but 19 (79%) did so at 150 mg per day. Five (12%) patients, all of whom developed a TR, had a partial response. Median progression-free survival (PFS) was 2.3 months (95% CI: 1.61, 4.14); median PFS was 3.5 months and 1.9 months, respectively, for patients who did and did not experience a TR (hazard ratio, 0.51; *P*=0.051). Neither rash severity nor response correlated with erlotinib exposure.

**Conclusion:**

Intrapatient dose escalation of erlotinib does not appreciably increase the propensity to experience a maximal level of tolerable skin toxicity, or appear to increase the anticancer activity of erlotinib in NSCLC.

Skin rash is the principal toxicity of both small molecule and antibody therapeutics targeting the epidermal growth factor receptor (EGFR), and retrospective analyses of a wide variety of studies have suggested that skin toxicity correlates with clinical benefit (Cohen *et al*, 2003; Perez-Soler *et al*, 2004; Saltz *et al*, 2004; Shepherd *et al*, 2005; Gibson *et al*, 2006; Jonker *et al*, 2007; Van Cutsem, 2007; Van Cutsem *et al*, 2007; Wacker *et al*, 2007). In the BR21 placebo-controlled phase III trial of erlotinib (Tarceva, OSI Pharmaceuticals Inc., Melville, NY, USA) in previously treated patients with non-small cell lung cancer (NSCLC), for example, the median survival for erlotinib-treated patients who did not experience skin toxicity was 3 months compared with 7 and 11 months for those with grade 1 or grade 2/3 skin toxicity, respectively (Wacker *et al*, 2007). Similar relationships have been observed in other clinical trials of erlotinib and other EGFR-targeting therapeutics, in a variety of malignancies (Soulieres *et al*, 2004; Gordon *et al*, 2005; Herbst *et al*, 2005; Berlin *et al*, 2007; Moore *et al*, 2007; Perez-Soler and Van Cutsem, 2007; O’Byrne *et al*, 2009; Verslype *et al*, 2009).

Since most patients with advanced NSCLC who are treated with erlotinib at its recommended dose of 150 mg per day do not experience dose-limiting skin toxicity (Hidalgo *et al*, 2001), it was reasoned that increasing the dose of erlotinib to that associated with maximal level of tolerable skin toxicity (i.e., a target rash (TR)) might increase the likelihood of observing anticancer activity. The present trial was designed to assess the feasibility of intrapatient erlotinib dose escalation to achieve a maximal level of tolerable skin toxicity and to evaluate the anticancer activity, pharmacokinetics (PK), and pharmacodynamic (PD) markers associated with erlotinib treatment at these increased doses.

## Materials and methods

### Patients

Patient eligibility included an age of at least 18 years, a confirmed diagnosis of stage 3B or 4 NSCLC, measurable disease, and treatment with at least one prior chemotherapy regimen in the advanced setting. Other relevant eligibility criteria included: a life expectancy of at least 12 weeks; Eastern Cooperative Group performance status of 0 or 1; and adequate haematopoietic, hepatic, and renal function. Patients with symptomatic or progressive central nervous system metastases, prior treatment with EGFR inhibitors; clinically relevant ophthalmologic abnormalities; and/or known hypersensitivity to the tetracyclines were excluded. The institutional review board/ethics committee of the participating institutions approved the study protocol and patients were required to give informed written consent.

### Trial design

The study was conducted according to an open-label, modified Simon two-stage design (Simon, 1989). In the initial stage, we assessed the feasibility of intrapatient dose escalation from 150 mg per day to a maximal level of tolerable skin toxicity, which was defined as the TR. This objective was determined to be feasible if patients developed a TR in the absence of non-cutaneous dose-limiting toxicity (DLT). If intrapatient dose escalation was feasible in at least five of the initial 15 patients, up to 31 additional patients were to be enrolled in the second stage of the study. Skin toxicity was graded according to the following scale that was based on patient tolerability:

- Grade 1 – a tolerable rash that did not require any symptomatic treatment.
- Grade 2 – a tolerable rash that required symptomatic treatment with minocycline, also defined as the TR.
- Grade 3 – an intolerable rash despite treatment with minocycline as specified in the following section; or a symptomatic rash associated with any of the following: opioid analgesics for pain and/or systemic corticosteroids for pruritus; desquamation/exfoliation of at least 25% of total body surface area; and/or generalised urticaria.

The starting dose for all patients was erlotinib 150 mg orally once daily for 21 days. In the absence of both a TR and non-cutaneous DLT, the dose was increased in each patient to 200 mg per day, and then in 25 mg per day increments every 14 days. Therefore, erlotinib doses were titrated in individual patients to achieve a grade 2 TR. Non-cutaneous DLT was defined as: grade 3 or 4 non-haematological toxicity, except for suboptimally managed nausea, vomiting, or diarrhea; grade 4 neutropenia lasting longer than 5 days; grade 3 or 4 neutropenia with fever; grade 3 or 4 infection; or a platelet count <25 000 *μ*l^−1^. Toxicities were graded according to the National Cancer Institute Common Toxicity Criteria (NCI CTC), Version 2.0, except for skin toxicity.

Doses were reduced in 25 mg per day decrements for toxicity. The initial systemic intervention for symptomatic skin toxicity was minocycline 100–150 mg twice daily for at least 7 days, and the initiation of treatment with minocycline for symptomatic rash was at the discretion of the investigator. The use of minocycline preemptively (i.e., to prevent the development of skin rash) was not allowed. In patients treated with minocycline, the erlotinib dose was not increased and could be held until the skin toxicity was felt to be tolerable, at which time, the same erlotinib dose could be resumed, and further dose escalation, as described previously, could be considered. Other interventions for skin toxicity, including short courses of topical corticosteroids, were at the investigators’ discretion. Patients continued erlotinib until disease progression, intolerable toxicity, or withdrawal of consent.

### Antitumour activity

Tumour assessments were performed using the Response Evaluation Criteria in Solid Tumours using computerised tomographic scanning or magnetic resonance imaging pretreatment and every 6–8 weeks during treatment (Therasse *et al*, 2000).

### Statistical analysis

Intrapatient dose escalation was considered feasible for patients who developed a TR without non-cutaneous DLT. In previous studies, ∼30% of the patients receiving erlotinib 150 mg per day developed NCI CTC grade 2 or 3 rash; therefore, the null and alternative hypotheses were that the feasibility rate was ⩽30% and ⩾50%, respectively (Shepherd *et al*, 2005; Perez-Soler and Van Cutsem, 2007; Van Cutsem *et al*, 2007). The optimal, modified two-stage Simon design tested these hypotheses with 0.05 probability of accepting a dose escalation strategy if the null hypothesis was true and 0.80 probability of rejecting the strategy if the alternative hypothesis was true. If dose escalation was feasible in at least five of the initial 15 patients, an additional 31 patients (46 total patients) were planned to be enrolled. The dose escalation strategy was to be declared feasible if 18 patients developed a TR.

Although this pilot study was not designed to demonstrate a statistically significant increase in antitumour activity compared with previous studies, a sample of 50 patients was estimated to provide a 70% power to detect an increase from 10% to 20% in response rate at the 5% level of significance, or 79% power at the 10% level of significance.

### PK assessments

Plasma samples were collected pretreatment, and 1, 2, 4, 6, 8, and 24 h on day 14 of cycle 1, as well as 14 days after any dose escalation. Samples were prepared and concentrations of erlotinib and its principal metabolite OSI-420 were measured by high-performance liquid chromatography as previously described (Hamilton *et al*, 2006). PK parameters were estimated by non-compartmental methods (WinNonlin v.3.1, Pharsight Corp., Mountain View, CA, USA).

### PD assessments

#### Tumour tissue

Paraffin embedded tumour blocks obtained at diagnosis and/or recurrence were collected for assessment of expression of EGFR, phosphorylated-EGFR (p-EGFR), extracellular signal-regulated kinase (ERK), and phosphorylated-ERK (p-ERK) using validated methods (EGFR PharmDX kit: Dako, Carpinteria, CA, USA). The study was performed before the significance of EGFR mutation status was fully appreciated and it was retrospectively concluded that the informed consent documents did not encompass patient approval for this assay.

#### Skin biopsy samples

Skin from affected and unaffected regions was biopsied pretreatment and following the first appearance of a TR.

#### Other PD assessments

Sebum and hair follicle samples were obtained for exploratory analyses, and results will be presented separately.

## Results

### Patients

Forty-two patients, whose relevant demographic characteristics are shown in Table 1, were enrolled between 2003 and 2005. Of note, 40 (95%) were either current or former smokers. All patients were evaluable for toxicity and anticancer activity.

### Erlotinib dose escalation and skin toxicity

During the first stage of the study, 10 of the 11 initial patients enrolled developed a TR, and therefore 31 additional patients were enrolled in the second stage of the study. Overall, 41 (98%) patients developed skin toxicity and 24 (57%) developed a TR, which exceeded the predefined 50% requirement for declaring the dose escalation scheme feasible. Nineteen (79%) patients developed a TR at the initial 150 mg per day dose level, whereas three (13%) and two (8%) patients developed a TR following escalation to 200 and 225 mg per day, respectively. Erlotinib doses were further escalated in 21 (88%) of the 24 patients who developed a TR.

Six patients experienced a grade 3 rash, which resulted in the reduction of doses to those associated with a TR. No patient was discontinued from the study due to skin toxicity. One patient did not develop a rash, and 16 patients never developed skin toxicity of sufficient severity to be considered a TR before the development of progressive disease.

Among the 38 (91%) patients in whom erlotinib doses were increased, 32 (76%) patients achieved a final dose exceeding 150 mg per day; doses were increased to 200 mg per day in 17 patients, 225–350 mg per day in 14 patients, and 475 mg per day in a single patient (Table 2). Four patients experienced early clinical disease progression and discontinued the study before developing TR.

### Anticancer activity

Five (12% 95% CI: 4.0–25.6) patients had a PR, which was achieved at a median maximal dose of 225 mg per day (range, 125–275 mg per day) (Table 3). The median duration of response was 11 months (range, 4 to 46^+^). The median time to initial response was 6 weeks (range, 5–76). The final daily dose among these patients ranged from 100 to 275 mg. All patients who experienced a PR had either grade 1 (three patients) or grade 2 (two patients) skin toxicity at the initial erlotinib dose and all achieved a TR during the study. Four of the responders were males; three were Caucasian, and two were Hispanic. All responders had non-squamous histology and were former smokers. EGFR immunostaining was positive in three responders, negative in one, with the last having an unknown status. Nineteen (45%) patients experienced stable disease (13 lasting at least 3 months), 10 of whom experienced a TR, as did 9 of 18 patients who had a best response of progressive disease.

The median progression-free survival (PFS) was 2.3 months (95% CI: 1.61–4.14) for all patients. PFS values were 12 months (range, 5.2 to 66+), 4.7 months (range, 1.2–14.0), and 1.4 months (range, 1.2–1.9) for patients who experienced a PR, SD, and progressive disease as their best response, respectively. The median PFS values for individuals who did and did not experience a TR were 3.5 months (95% CI: 1.61, 6.97) and 1.9 months (95% CI: 1.54, 4.14), respectively. There was a 49% reduction in the risk of disease progression or death in patients who had TR (hazard ratio, 0.51% 95% CI: 0.25–1.02; *P*=0.051).

### Toxicity

Toxicities as a function of dose are displayed in Table 4. The principal toxicity of erlotinib in this study was skin rash, predominately involving the face, neck, and upper torso and characterised as erythemateous, maculopapular, acneiform, and/or pustular. In 12 (29%) patients, the rash resembled rosacea, whereas urticaria and desquamation were the dominant features in 11 (26%) and 16 (38%) patients, respectively. Symptoms associated with skin toxicity, which were generally mild to modest in severity, included pruritus in 28 (67%) patients and pain in 10 (24%) patients. Medication to ameliorate symptoms were administered to 32 (76%) of patients. The use of minocycline and topical corticosteroids was documented in 60% and 38% of patients, respectively.

Seven patients experienced non-cutaneous DLT, all of whom required dose reduction and symptomatic treatment. These dose-limiting events included grade 3 fatigue (three patients), as well as grade 3 headache, grade 3 creatinine elevation, grade 3 paronychia, and grade 3 anorexia (one patient each). One patient discontinued treatment due to protracted grade 3 anorexia and grade 2 nausea. The most common toxicities included nausea (52% of patients), vomiting (36%), diarrhea (79%), and stomatitis (33%), but these events were well managed with routine supportive measures. Fatigue and paronychia were also noted in 40% and 26% of the patients, respectively. The frequency, but not the severity of diarrhea, nausea, fatigue, and anorexia, was greater in patients treated at the higher erlotinib dose levels.

### Dermatological evaluations

Skin biopsies at the time of first appearance of TR were performed in 23 (54%) patients. The principal histopathological findings included altered terminal differentiation of keratinocytes, distorted pilosebaceous units in the interfollicular epidermis, and mononuclear cell infiltration associated with follicular destruction. The pathological findings will be reported separately.

### Pharmacokinetics

**Figure 1:**
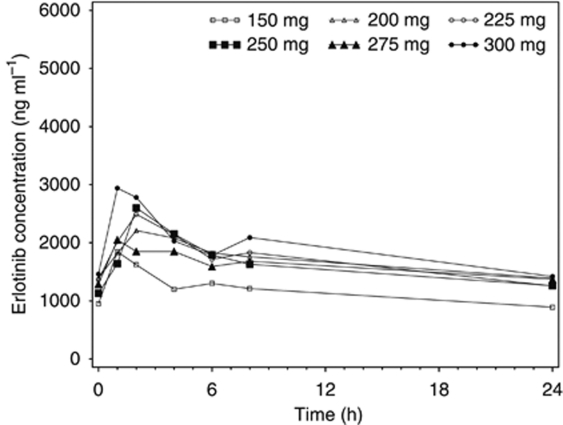
Mean erlotinib plasma concentration–time profiles at highest dose attained.

Thirty-one (74%) of 42 patients had PK analysis at erlotinib doses exceeding 150 mg per day. PK samples were available at 200 mg in 25 patients, 300 mg in 5, 325 and 350 mg in 2, and 375, 400, 425, 450 and 475 mg in 1 patient each. The principal PK parameters for erlotinib and OSI-420 as a function of erlotinib dose level are summarised in Table 5. High interpatient and intrapatient variability for both erlotinib and OSI-420 was observed. A Spearman rank correlation analysis of the relationships between dose and both AUC_0–24_ and *C*_max_ (across the dose range with sufficient number of observations, 150–300 mg) did not reveal any significant deviation from dose proportionality; however, the interpretation of such analyses is limited by the high intrapatient variability and the small number of patients at higher dose levels. Mean erlotinib plasma concentration–time profiles at the highest dose attained are illustrated in Figure 1.

No significant relationships between erlotinib exposure and relevant patient characteristics were observed. The single exception was smoking status, in which the erlotinib AUC_0–24_ was significantly lower in smokers compared with former or never smokers (*P*-values 0.03 and 0.004, respectively); however, the numbers of current and never smokers were small. Rash severity did not relate to erlotinib exposure (determined after 14 days of each escalation) nor did best response relate to PK parameters (AUC_0–24_ and *C*_max_) reflecting exposure to erlotinib and OSI-420.

### Pharmacodynamics

Among the 34 patients evaluable for EGFR expression on archival tumour blocks, 29 (85%) had EGFR immunostaining in at least 10% of tumour cells. The intensity of EGFR immunostaining did not relate to the achievement of a TR, response, nor PFS. Although a trend towards reductions in phosphorylated-EGFR/EGFR protein activity compared with pretreatment levels was seen in a larger proportion in patients who developed the TR compared with patients who did not, no definitive conclusions can be drawn regarding these skin PD studies and the severity of rash and/or response due to the small number of samples available (data not shown).

## Discussion

This study demonstrated that the dose escalation of erlotinib above its recommended dose of 150 mg per day to a maximal level of tolerable skin toxicity is feasible but does not seem to result in increased antitumour activity in this patient population (Shepherd *et al*, 2005; Ardavanis *et al*, 2008; Felip *et al*, 2008; Hesketh *et al*, 2008; Spiegel *et al*, 2008; Tiseo *et al*, 2009). Erlotinib doses could not be increased above the dosing range of 250–300 mg per day in most patients, principally due to intolerable skin toxicity despite various supportive measures, particularly treatment with minocycline and topical corticosteroids. Although dose escalation of erlotinib above 150 mg per day was feasible, 19 (79%) of 24 patients who achieved a TR did so after a short period of treatment at the initial erlotinib dose level of 150 mg per day, and only five (21%) of the patients who ultimately experienced a TR did so after dose escalation. These findings suggest that further erlotinib dose escalation above 150 mg per day does not increase the propensity of achieving a TR in most individuals who have the potential to do so, and that 150 mg per day is an adequate erlotinib dose if the objective is to achieve a TR. In 22 patients, erlotinib doses were increased above the dose that was initially associated with the development of a TR. However, treatment at higher erlotinib doses was associated with a higher rate of mild-to-moderate fatigue, diarrhea, nausea, vomiting, and stomatitis than reported for the approved 150 mg per day dose (Shepherd *et al*, 2005).

A notable increase in anticancer activity was not observed in this unselected patient population. For example, median PFS and ORR values were 2.2 months (95% CI: 1.9–2.9 months) and 8.9% (95% CI: 6.4–12%), respectively, in the BR.21 study (Shepherd *et al*, 2005), compared with 2.3 months (95% CI: 1.61–4.14) and 12% (95% CI: 4.0–25.6), in the present study. Although these results suggest that the dose escalation approach used in this study is unlikely to confer incremental activity in this patient population, the existence of a dose–activity relationship, which may have been demonstrated using alternate approaches such as treatment with higher erlotinib doses from the outset, cannot be ruled out. The dose escalation scheme in this study was conservative, and disease progression was rapid in most patients, thereby precluding a meaningful duration of treatment at higher doses in a sufficient number of patients. However, all patients who achieved an objective response experienced a TR and it is noteworthy that the median time to objective response in these patients was relatively brief. Furthermore, both the initial and final erlotinib doses associated with these objective responses, as well as the TR, were not much higher than the starting dose of 150 mg per day. These findings suggest that the propensity to experience an objective response is more likely to be linked to factors inherent to the individual patient and the tumour rather than dose and related factors such as drug exposure. Finally, four patients in our study underwent dose reduction to 125 or 100 mg erlotinib after developing intolerable rash at 150 mg, and they continued on treatment for a median of 84 days (range, 14–153). These findings, together with the reports of efficacy of lower doses of erlotinib in patients with known EGFR mutations suggest that, in the presence of rash, dose reductions to ‘subtherapeutic’ levels remain effective and may prevent unnecessary early treatment termination (Lind *et al*, 2009).

The principal objective of the present study was to determine the antitumour activity of erlotinib in previously treated NSCLC patients by achieving maximal rash based on numerous retrospective analyses of clinical trial data (Cohen *et al*, 2003; Perez-Soler *et al*, 2004; Saltz *et al*, 2004; Gibson *et al*, 2006; Berlin *et al*, 2007; Jonker *et al*, 2007; Perez-Soler and Van Cutsem, 2007; Van Cutsem, 2007; Van Cutsem *et al*, 2007; Wacker *et al*, 2007).

The hypothesis was based on the notion that skin toxicity may be a surrogate for the inhibition of EGFR TK phosphorylation in the cancer itself. However, since it is now recognised that the most robust tumour responses noted with EGFR inhibitors occur in patients with activating EGFR mutations (Lynch *et al*, 2004), it is not surprising that dose escalation of erlotinib to the maximal dose associated with a TR did not result in an increased response rate in this unselected patient population. Unfortunately, tumour tissue was not assessed for EGFR mutation analysis due to the absence of informed consent for mutational analyses as the trial was conducted before significance of EGFR mutations were reported. EGFR expression in tumour tissue in this trial was determined by immunohistochemistry; however, the optimal biomarker for the selection of patients with NSCLC for treatment with EGFR inhibitors remains to be determined. Recent studies have determined that the EGFR gene copy number (as determined by FISH) in addition to the EGFR mutation status may also be important predictors to response to small-molecule inhibitors of EGFR (Hirsch *et al*, 2008). Nevertheless, it is possible, albeit unlikely based on the results of the present study, that higher doses of erlotinib may result in more potent inhibition of wild-type EGFR, whose overexpression/activation may also play a role in the pathogenesis of NSCLC (Fujimoto *et al*, 2005). Larger randomised trials (i.e., 150 mg per day as a fixed dose *vs* rapidly escalating doses of erlotinib) in a selected population for the relevant target and with adequate patient stratification (i.e., by gender, histology, smoking history, EGFR amplification/mutation, etc.) would be needed to rigorously address this hypothesis.

There were no relationships between erlotinib PK parameters reflecting drug exposure and clinical activity in this study, which is not surprising. Indeed, the large intrapatient variability of the erlotinib PK observed in this and other studies, as well as the relatively small size of the trial, may have contributed negatively, in part, to this observation (Soulieres *et al*, 2004; Herbst *et al*, 2005; Perez-Soler and Van Cutsem, 2007). The lack of relationships between PK parameters of drug exposure with toxicity and clinical activity also underscores the importance of tumour biology and host features relative to dose and drug exposure in the development of toxicity and clinical benefit in this disease setting. Similarly, no relationship between the PK parameters of OSI-420, the main active metabolite of erlotinib and clinical activity were observed.

In conclusion, intrapatient dose escalation of erlotinib above 150 mg to achieve a TR was feasible, but most patients who developed TR did so at the initial dose and this dose escalation scheme did not result in increased anticancer activity in previously treated, unselected NSCLC patients compared with historical data in patients treated with a standard dosing regimen. In addition, treatment with erlotinib at higher doses resulted in increased non-cutaneous toxicities, which may have negatively impacted on the quality of life in some patients. Therefore, dose escalation of erlotinib above the approved dose of 150 mg is not recommended for use in this disease setting.

## Figures and Tables

**Table 1:** Patient characteristics (*n*=42)

**Characteristic**	**Number (%)**
Male/female (%)	14 (33%)/28 (67)
	
*Age*	
Median (range), years	63 (41–78)
	
*Race/ethnicity*	
Caucasian	34 (81)
Hispanic	5 (12)
African-American	2 (5)
Asian	1 (2)
	
*ECOG performance status*	
0	11 (26)
1	30 (71)
2	1 (2)
	
*No. of prior chemotherapy regimens*	
1	19 (45)
2	13 (31)
>2	10 (24)
	
*Smoking status*	
Never smokers	2 (5)
Past history of smoking	35 (83)
Current smokers	5 (12)
	
*Histologic subtype*	
Adenocarcinoma	23 (56)
Squamous cell carcinoma	11 (26)
Undifferentiated large cell	4 (10)
Mixed histology	2 (5)
Other	2 (5)
	
*EGFR IHC at diagnosis (>10% staining)*	
Positive	29 (68)
Negative	5 (12)
Unknown	8 (20)

Abbreviations: ECOG=Eastern Cooperative Oncology Group; EGFR=epidermal growth factor receptor; IHC=immunohistochemistry.

**Table 2:** Erlotinib dose escalation

	**Maximum dose level**		**Final dose level**	
**Dose level**	**Number of patients**	**Median days of treatment (range)**	**Number of patients**	**Median days of treatment (range)**
100	—	—	2 (5%)	81 (21–140)
125	—	—	2 (5%)	84 (14–153)
150	4 (10%)	22 (12–49)	6 (14%)	63 (42–119)
175	—	—	2 (5%)	120 (30–210)
200	13 (31%)	14 (2–18)	15 (36%)	15 (2–342)
225	9 (21.5%)	14 (8–27)	5 (12%)	14 (8–14)
250	7 (17%)	8 (5–50)	4 (10%)	14 (5–50)
275	1 (2%)	26	2 (5%)	322 (26–618)
300	5 (12%)	51 (9–93)	2 (5%)	65 (51–78)
350	1 (2%)	14	1 (2%)	14
425	1 (2%)	1		
475	1 (2%)	41	1 (2%)	41

**Table 3:** Antitumour activity as a function of target rash

	**Objective responses (CR+PR)**			**Disease control (CR+PR+SD)**		
**Patient population**	**No. of patients with PR+CR/total no. of patients**	**Response rate (95% CI)**	**Fisher's exact test *P*-value**	**No. of patients with CR+PR+s.d./total no. of patients**	**Response rate (95% CI)**	**Fisher's exact test *P*-value**
All patients	5/42	11.9 (4.0, 25.6)		24/42	57.1 (41.0, 72.3)	
Target rash feasible	5/24	20.8 (7.1, 42.2)	0.06	15/24	62.5 (40.6, 81.2)	0.53
Target rash not feasible	0/18	0.0 (N/A)		9/18	50.0 (26.0, 74.0)	

Abbreviations: CR=complete response; CI=confidence interval; PR=partial response; SD=stable disease; N/A=not applicable.

**Table 4:** Erlotinib-related toxicities (worst grade per patient) as a function of dose

	**Erlotinib dose**									
	**100**–**150 mg (*N***=**4)**		**175**–**200 mg (*N***=**13)**		**225**–**275 mg (*N***=**18)**		⩾**300 mg (*N***=**7)**		**Total (*N***=**42)**	
	**Grade**		**Grade**		**Grade**		**Grade**		**Grade**	
	**Any**	**3**–**4**	**Any**	**3**–**4**	**Any**	**3**–**4**	**Any**	**3**–**4**	**Any**	**3–4**
**Toxicity**	***n* (%)**	***n* (%)**	***n* (%)**	***n* (%)**	***n* (%)**	***n* (%)**	***n* (%)**	***n* (%)**	***n* (%)**	***n* (%)**
Diarrhea	4 (100)	0 (0)	9 (69)	0 (0)	13 (72)	1 (6)	7 (100)	0 (0)	33 (79)	1 (2)
Nausea	3 (75)	0 (0)	5 (38)	0 (0)	9 (50)	0 (0)	5 (71)	0 (0)	22 (52)	0 (0)
Vomiting	2 (50)	0 (0)	5 (38)	0 (0)	5 (28)	0 (0)	3 (43)	0 (0)	15 (36)	0 (0)
Stomatitis	2 (50)	0 (0)	5 (38)	0 (0)	6 (33)	0 (0)	1 (14)	0 (0)	14 (33)	0 (0)
Fatigue	3 (75)	0 (0)	4 (31)	1 (8)^a^	6 (33)	2 (11)^a^	4 (57)	0 (0)	17 (40)	3 (7)
Alopecia	0 (0)	0 (0)	0 (0)	0 (0)	4 (22)	0 (0)	2 (29)	0 (0)	6 (14)	0 (0)
Skin fissures	1 (25)	0 (0)	0 (0)	0 (0)	2 (11)	1 (6)	2 (29)	0 (0)	5 (12)	1 (2)
Anorexia	2 (50)	0 (0)	0 (0)	0 (0)	6 (33)	1 (6)^a^	3 (43)	0 (0)	11 (26)	1 (2)
Paronychia	0 (0)	0 (0)	0 (0)	0 (0)	3 (17)	0 (0)	2 (29)	1 (14)^a^	5 (12)	1 (2)
Epistaxis	0 (0)	0 (0)	0 (0)	0 (0)	3 (17)	0 (0)	2 (29)	0 (0)	5 (12)	0 (0)
Headache	0 (0)	0 (0)	0 (0)	0 (0)	1 (6)	1 (6)^a^	0 (0)	0 (0)	1 (2)	1 (2)
Creatinine	0 (0)	0 (0)	1 (8)	1 (8)^a^	0 (0)	0 (0)	0 (0)	0 (0)	1 (2)	1 (2)

Abbreviation: *N*=number.

a Non-cutaneous dose limiting toxicity reported.

**Table 5:** Relevant pharmacokinetic parameters for erlotinib and OSI-420 as a function of erlotinib dose

		**Erlotinib**				**OSI-420**			
**Erlotinib dose (mg** **m**^−**2**^**)**	**No. of patients**	***C*_max_** **(*μ*g ml^−1^)**	*T*_max_ (h)***	**AUC_0–24_** **(*μ*g h ml^−1^)**	**C_24_** **(*μ*g ml^−1^)**	***C*_max_** **(*μ*g ml^−1^)**	***T*_max_** (h)***	**AUC_0–24_** **(*μ*g h ml^−1^)**	**C_24_** **(*μ*g ml^−1^)**
150	13	1.91 (0.787–3.47)	2.0 (1.00–23.9)	28.6 (9.72–49.7)^a^	0.890 (0.0292–3.47)^a^	0.165 (0.0452–0.568)	4.0 (1.05–23.9)	2.56 (0.599–10.5)^a^	0.0926 (0.00355–0.430)
200	25	2.65 (1.09–5.68)	2.0 (1.00–8.05)	42.8 (13.3–97.7)^b^	1.35 (0.163–3.24)^b^	0.278 (0.0617–0.864)	2.0 (1.00–24.0)	4.55 (0.996–19.2)	0.129 (0.0113–0.774)
225	18	2.54 (1.11–4.74)	2.0 (0.930–8.00)	38.5 (19.9–83.1)	1.26 (0.311–2.92)	0.260 (0.116–0.908)	2.0 (0.930–8.00)	4.10 (1.57–19.7)	0.125 (0.0220–0.785)
250	11	2.82 (1.34–3.30)	2.1 (1.00–6.00)	38.3 (15.5–60.3)	1.26 (0.132–2.19)	0.219 (0.119–0.760)	4.0 (1.00–8.00)	3.32 (1.52–16.4)	0.0915 (0.0118–0.636)
275	9	2.19 (1.30–3.92)	1.0 (1.00–8.02)	38.2 (15.9–66.6)	1.37 (0.169–4.87)	0.223 (0.107–0.929)	2.0 (1.00–8.02)	3.62 (1.51–15.7)	0.0896 (0.0164–0.591)
300	5	2.94 (1.42–4.48)	2.0 (1.00–8.00)	47.5 (13.8–52.7)	1.42 (0.214–1.90)	0.273 (0.133–0.346)	2.0 (1.00–2.03)	4.23 (1.38–6.32)	0.123 (0.0203–0.194)
325	2	2.80 (1.79, 3.80)	1.5 (2.00, 1.00)	37.1 (19.4, 54.7)	0.672 (0.224, 1.12)	0.344 (0.222, 0.426)	1.5 (2.00, 1.00)	4.18 (2.26, 6.09)	0.0708 (0.0206, 0.121)
350	2	1.78 (2.18, 1.37)	3.5 (1.03, 6.00)	23.7 (24.3, 23.0)	0.579 (0.452, 0.706)	0.169 (0.243, 0.0945)	2.0 (2.00, 2.03)	2.33 (2.94, 1.72)	0.0478 (0.0440, 0.0516)
375	1	2.77	1.0	42.4	1.30	0.253	1.0	3.80	0.123
400	1	4.48	2.0	59.5	1.74	0.454	2.0	6.84	0.188
425	1	2.78	4.0	47.7	1.54	0.293	2.0	5.02	0.152
450	1	4.10	8.0	79.6	2.40	0.489	4.0	8.78	0.267
475	1	2.2	4.2	42.1	1.53	0.177	4.2	3.30	0.112

Abbreviations: *C*_max_=maximum plasma concentration; *T*_max_=time to maximum concentration; AUC_0–24_=area under the curve from time 0 to 24 h; C_24_=plasma concentration at 24 h after the most recent dose.

a *n*=12.

b *n*=24.

^***^T_max_ is summarised as median (range).
